# Direct Sequencing of *Cryptosporidium* in Stool Samples for Public Health

**DOI:** 10.3389/fpubh.2019.00360

**Published:** 2019-12-11

**Authors:** Arthur Morris, Guy Robinson, Martin T. Swain, Rachel M. Chalmers

**Affiliations:** ^1^Institute of Biological, Environmental & Rural Sciences, Aberystwyth University, Aberystwyth, United Kingdom; ^2^Cryptosporidium Reference Unit, Public Health Wales Microbiology, Singleton Hospital, Swansea, United Kingdom; ^3^Swansea University Medical School, Swansea, United Kingdom

**Keywords:** cryptosporidium, public health, genotyping, genome, sequencing, multiplicity of infection

## Abstract

The protozoan parasite *Cryptosporidium* is an important cause of diarrheal disease (cryptosporidiosis) in humans and animals, with significant morbidity and mortality especially in severely immunocompromised people and in young children in low-resource settings. Due to the sexual life cycle of the parasite, transmission is complex. There are no restrictions on sexual recombination between sub-populations, meaning that large-scale genetic recombination may occur within a host, potentially confounding epidemiological analysis. To clarify the relationships between infections in different hosts, it is first necessary to correctly identify species and genotypes, but these differentiations are not made by standard diagnostic tests and more sophisticated molecular methods have been developed. For instance, multilocus genotyping has been utilized to differentiate isolates within the major human pathogens, *Cryptosporidium parvum* and *Cryptosporidium hominis*. This has allowed mixed populations with multiple alleles to be identified: recombination events are considered to be the driving force of increased variation and the emergence of new subtypes. As yet, whole genome sequencing (WGS) is having limited impact on public health investigations, due in part to insufficient numbers of oocysts and purity of DNA derived from clinical samples. Moreover, because public health agencies have not prioritized parasites, validation has not been performed on user-friendly data analysis pipelines suitable for public health practitioners. Nonetheless, since the first whole genome assembly in 2004 there are now numerous genomes of human and animal-derived cryptosporidia publically available, spanning nine species. It has also been demonstrated that WGS from very low numbers of oocysts is possible, through the use of amplification procedures. These data and approaches are providing new insights into host-adapted infectivity, the presence and frequency of multiple sub-populations of *Cryptosporidium* spp. within single clinical samples, and transmission of infection. Analyses show that although whole genome sequences do indeed contain many alleles, they are invariably dominated by a single highly abundant allele. These insights are helping to better understand population structures within hosts, which will be important to develop novel prevention strategies in the fight against cryptosporidiosis.

## Introduction

**Figure 1:**
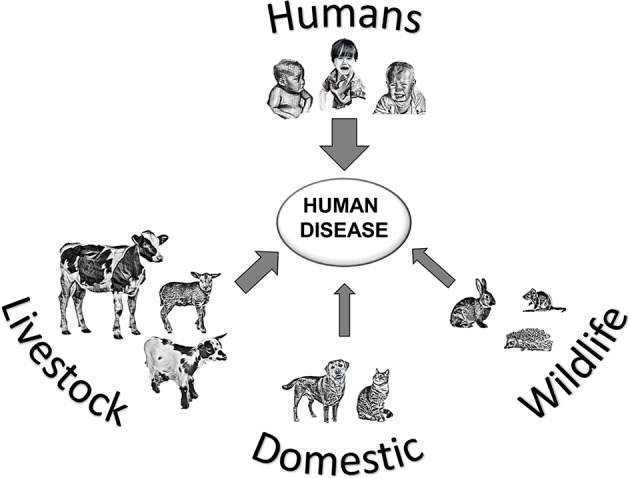
Transmission of *Cryptosporidium* spp. leading to human cryptosporidiosis, arrow thickness represents likely global importance of source hosts.

**Figure 2:**
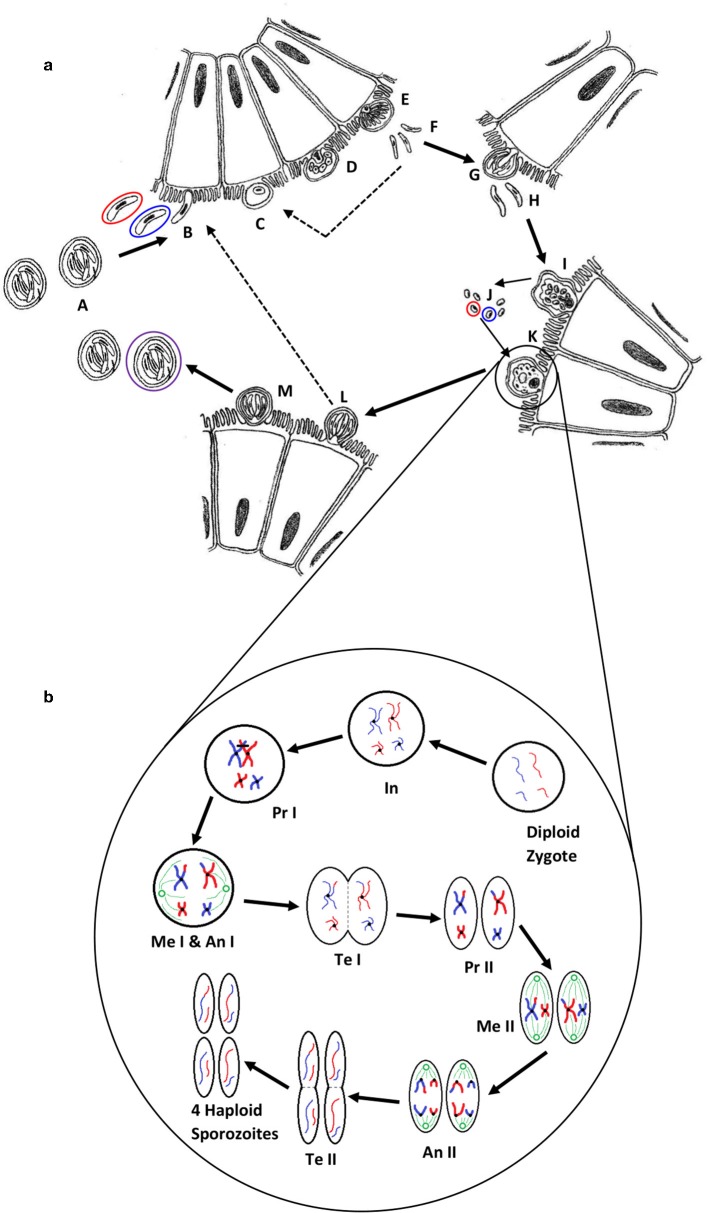
**(a)** The life cycle of *Cryptosporidium* (1). Oocysts (A) are ingested by the host, most likely as a mixed population of different genotypes; haploid sporozoites (B) (variants are represented by red and blue) excyst and invade the brush border of epithelial cells; each sporozoite develops into a haploid trophozoite with a prominent nucleus (C); the trophozoite undergoes merogony by mitosis to form a type I meront (D,E); up to eight haploid merozoites (F) are released, invade another cell and undergo merogony again to form either further type I meronts (dotted line) or type II meronts (G), which release four haploid merozoites (H) and form either microgamonts (I) that become multinucleate and mature to form multiple haploid microgametes (J) by mitosis, or a haploid macrogamont (K). Microgamonts are released and potentially each fertilize a macrogamont to form a diploid zygote which undergoes sporogony by meiosis to produce either thin-walled oocysts (L) containing four haploid sporozoites that can autoinfect the host (dotted line), or thick-walled oocysts (M) that are shed in the feces ready to transmit four haploid sporozoites to a new host (the purple circle represents an oocyst that is the product of fertilization between the red and blue genotypes). **(b)** A simplified schematic of genetic recombination in *Cryptosporidium*, potentially generating variation between sporozoites within oocysts. In a mixed infection population, different fertilization scenarios potentially occur—between the same genotypes (resulting in identical daughter sporozoites) or between different genotypes, as in the example shown, that result in a variety of outcomes depending on the random genetic exchange, or lack of, that occurs during meiosis. For simplicity only two example chromosomes are shown with DNA from different genotypes represented by blue and red. The diploid zygote contains duplicate pairs of chromosomes, one set from each parent cell; during interphase (In) the DNA in each chromosome is replicated to produce two identical sister chromatids held together with a centromere; in prophase I (Pr I) the chromosomes start to condense and pair up with the homologous chromosome from the other parent cell, and cross-over can occur resulting in an genetic exchange; during metaphase I (Me I) the paired chromosomes line up along the center of the cell and microtubules connect the centromeres to the centrosomes (shown in green); during anaphase I (An I) each complete set of chromosomes (still paired as sister chromatids) are pulled toward each centrosome—the chromosomes from either parent are randomly combined at this phase introducing a further opportunity for recombination (a blue and a red chromosome are drawn to each centrosome in this example); in telophase I (Te I) the chromosomes start to unravel and cytokinesis starts to split the cell into two, resulting in two haploid cells; in prophase II (Pr II) the chromosomes condense again; during metaphase II (Me II) the chromosomes line up along the center of the cells and microtubules connect the centromeres to the centrosomes; this time during anaphase II (An II) the sister chromatids are separated and pulled apart toward the centrosomes, creating new daughter chromosomes; finally in telophase II (Te II) the chromosomes unravel and cytokinesis starts to split the cells, which in the case of this example due to the crossover event in prophase I, results in four genetically different haploid sporozoites. Depending upon whether random genetic exchanges take place between chromosomes from different genotype parents (either in prophase I or anaphase I) the resulting haploid sporozoites can either be all different, two pairs of identical sporozoites that are different from each parent, or two pairs of identical sporozoites that are the same as the two parents.

The parasite *Cryptosporidium* is a protozoan that occurs worldwide, and can cause the diarrheal disease cryptosporidiosis in humans and animals (Figure 1). The life cycle of *Cryptosporidium* (Figure 2a) (1) is completed within a single host. Both the asexual phase, and the production of thin-walled oocysts that enable autoinfection, mean the numbers of parasites are increased from possibly single figures in the initial infection, to result in clinically significant infections and the shedding of vast numbers of oocysts in feces (2). These shed oocysts have thick walls, conferring protection for the four infective sporozoites contained within, and enabling long-term survival, environmental transmission, and resistance to commonly used disinfectants including chlorine (3, 4). This means that, in addition to the variety of hosts that act as direct sources of infection (Figure 1; Table 1), contaminated food, water, or environmental vehicles are involved in transmission and need to be considered and investigated for effective disease control and prevention of outbreaks of cryptosporidiosis (5).

**Table 1:** *Cryptosporidium* species, their major hosts, oocyst dimensions, reported human infectivity and availability of genome data.

***Cryptosporidium* species**	**Mean oocyst dimensions (μm)**	**Major host(s)**	**Infections reported in humans**	**Genomes available** **(accession number)**
*C. alticolis*	5.4 × 4.9	Voles	No	No
*C. apodemi*	4.2 × 4.0	Mice	No	No
*C. andersoni*	7.4 × 5.5	Cattle	Yes (rarely)	PRJNA354069
*C. avium*	6.3 × 4.9	Birds	No	No
*C. baileyi*	6.2 × 4.6	Birds	No	PRJNA222835
*C. bovis*	4.9 × 4.6	Cattle	Yes (rarely)	No
*C. canis*	5.0 × 4.7	Canids	Yes (occasionally)	No
*C. cuniculus*	5.6 × 5.4	Lagomorphs, Humans	Yes (occasionally)	PRJNA315496
*C. ditrichi*	4.7 × 4.2	Mice	Yes (rarely)	No
*C. ducismarci*	5.0 × 4.8	Tortoises	No	No
*C. erinacei*	4.9 × 4.4	Hedgehogs	Yes (rarely)	No
*C. fayeri*	4.9 × 4.3	Marsupials	Yes (rarely)	No
*C. felis*	4.6 × 4.0	Felids	Yes (occasionally)	No
*C. fragile*	6.2 × 5.5	Toads	No	No
*C. galli*	8.3 × 6.3	Birds	No	No
*C. homai*	Data not available	Guinea Pigs	No	No
*C. hominis*	4.9 × 5.2	Humans	Yes (commonly)	PRJEB10000 PRJNA13200 PRJNA252787 PRJNA222836 PRJNA222837 PRJNA307563 PRJNA253838 PRJNA253839 PRJNA253834
*C. huwi*	4.6 × 4.4	Fish	No	No
*C. macropodum*	5.4 × 4.9	Marsupials	No	No
*C. meleagridis*	5.2 × 4.6	Birds, mammals	Yes (occasionally)	PRJNA222838 PRJNA315503 PRJNA315502
*C. microti*	4.3 × 4.1	Voles	No	No
*C. molnari*	4.7 × 4.5	Fish	No	No
*C. muris*	7.0 × 5.0	Rodents	Yes (rarely)	PRJNA32283 PRJNA19553
*C. occultus*	5.2 × 4.9	Rodents	Yes (rarely)	No
*C. parvum*	5.0 × 4.5	Mammals	Yes (commonly)	PRJNA144 PRJNA320419 PRJNA439211 PRJNA253848 PRJNA253843 PRJNA253845 PRJNA253836 PRJNA253840 PRJNA253846 PRJNA253847 PRJNA320419 PRJNA315506 PRJNA437480 PRJNA315504 PRJNA315508 PRJNA315507 PRJNA315505 PRJNA13873
*C. proliferans*	7.7 × 5.3	Rodents, maybe Equids	No	No
*C. proventriculi*	7.4 × 5.7	Birds	No	No
*C. rubeyi*	4.7 × 4.3	Squirrels	No	No
*C. ryanae*	3.7 × 3.2	Cattle	No	No
*C. scrofarum*	5.2 × 4.8	Pigs	Yes (rarely)	No
*C. serpentis*	6.2 × 5.3	Reptiles	No	No
*C. suis*	4.6 × 4.2	Pigs	Yes (rarely)	No
*C. testudinis*	6.4 × 5.9	Tortoises	No	No
*C. tyzzeri*	4.6 × 4.2	Rodents	Yes (rarely)	No
*C. ubiquitum*	5.0 × 4.7	Mammals	Yes (occasionally)	PRJNA534291 PRJNA315509 PRJNA315510
*C. varanii*	4.8 × 4.7	Reptiles	No	No
*C. viatorum*	5.4 × 4.7	Humans, Rodents	Yes (occasionally)	PRJNA492837
*C. wrairi*	5.4 × 4.6	Guinea Pigs	No	No
*C. xiaoi*	3.9 × 3.4	Sheep, Goats	No	No

Human cryptosporidiosis is usually a gastrointestinal disease, although there is some evidence for respiratory cryptosporidiosis in some populations (6). Symptoms ranging from mild to severe depending upon a number of factors, including the host's age, immune status, nutrition, genetics, and the site of infection, as well as the infecting species and variant of *Cryptosporidium* (7–9). Clinical symptoms include diarrhea, abdominal pain, vomiting, nausea, and low-grade fever, which, although prolonged (2 weeks is not unusual) are generally self-limiting in immune competent hosts. However, infection can be more problematic and even life-threatening in some severely immunocompromised individuals, and in malnourished young children (10). There are few options for treatment or prevention. Recent studies have shown that in some low-resource countries, where access to safe drinking water, sanitation, hygiene, and healthcare is often poor, *Cryptosporidium* is one of the most important causes of moderate-to-severe diarrheal disease and death in young children (11, 12). Furthermore, long-term effects of infection such as malnutrition, growth, and cognitive deficits have been described, highlighting the socio-economic impact on the adverse outcomes of infection (10). A vicious cycle of malnutrition and diarrhea can become established with detrimental effects on these societies (13). For these reasons, *Cryptosporidium* was included in the World Health Organization's Neglected Diseases Initiative in 2004 (14), which served to raise awareness of the need for international and national investments in prevention and control.

Thirty-nine species of *Cryptosporidium* have been described at the time of writing (Table 1), but not all cause human disease. The vast majority of human cryptosporidiosis is caused by the zoonotic species *Cryptosporidium parvum* or anthroponotic *Cryptosporidium hominis*, with multiple variants that can cause varying severity of symptoms. The diagnostic target of laboratory tests, and those used to detect *Cryptosporidium* in water, is the oocyst, using stained microscopy or immunologically-based assays, or the sporozoite DNA. Routinely applied tests are not able to differentiate species, and molecular methods are needed to investigate true relationships between infections and contaminants and thus elucidate the complex transmission of *Cryptosporidium*. A range of samples need to be investigated, from feces (e.g., stools, diapers, livestock dung, manure, slurry, runoff, and wild life droppings), to contaminated water and food, but these present challenges to detection and genotyping. At present, amplification by culture is not an option in this context, and finding oocyst targets, which may be in low concentration in the sample matrix, can be a hit-and-miss affair. Recent advances in molecular methods generally, and particularly in genomics, have increased the amount of data available particularly on the major pathogenic *Cryptosporidium* species (Table 1). Continued generation and accessibility of genomic data will potentially improve the public health response to cryptosporidiosis by identifying new targets for incorporation into diagnostic and genotyping assays (15). Putative virulence and host adaption factors have been proposed (16), and potential chemotherapeutic targets and vaccine candidates are being sought (10, 17) and identified [e.g., (18)].

## Introduction to *Cryptosporidium* Genotyping

To identify *Cryptosporidium* species, genotyping was undertaken initially using conventional PCR combined with either restriction fragment length polymorphism (RFLP) or Sanger sequence analysis, most commonly of the 18S rRNA gene (19). The 18S rRNA gene includes conserved regions interspersed with highly polymorphic regions and is currently considered to provide the definitive sequences for discriminating *Cryptosporidium* species. It is present in multiple copies (5 per sporozoite; 20 per oocyst) facilitating the development of sensitive assays, which is especially important for testing samples such as water where small (but potentially significant) numbers of oocysts may be present. Species-level genotyping has provided improved understanding of human epidemiology in some countries, streamlined by the use of real-time PCR (see below). DNA extraction methods from stool and gene targets have been reviewed in detail by Khan et al. (17).

Beyond the species-level, Sanger sequencing part of the *gp60* gene is most commonly used for further discriminating some *Cryptosporidium* species, including *C. parvum* and *C. hominis* (19–21). The *gp60* gene is hypervariable both between and within *Cryptosporidium* species, and the presence of a highly variable serine repeat region in most species enables further discrimination (19). For nomenclature of *gp60* subtypes, the reader is referred to a review of molecular epidemiologic tools by Xiao and Feng (19). The use of this locus as a subtyping marker has been questioned as it is associated with host cell invasion, and therefore can be considered a virulence factor under selective pressure. Nevertheless, as shown below, it may still be an appropriate target for interrogation as a phenotype determining biomarker. Another issue arises from the use of a single locus; this may not be appropriate due to the genetic recombination that occurs within *Cryptosporidium* populations during the sexual stage of the life-cycle (Figure 2b). Whilst not likely or expected between different species, this may occur in populations of mixed subtypes of the same species (22–25). This necessitates the investigation of multiple loci to reveal a more accurate estimate of diversity and population structure (19, 26), and would confer greater discrimination for characterization of isolates (26, 27).

The reality is that genotyping tools are not currently widespread in their application for public health purposes and in most countries *Cryptosporidium* is under-diagnosed and isolates are not characterized (28). In low-resource countries where surveillance data are lacking, research studies have found that *C. hominis* or human-adapted *C. parvum* subtypes predominate (29, 30). *C. parvum* can also be the main species detected in some urban settings with no animals close to residences, further suggesting anthroponotic rather than zoonotic transmission (29). These findings indicate that measures to improve sanitation and hygiene would have greatest impact in these settings. Not only is there a high prevalence of *Cryptosporidium* in these populations, but there is also greater diversity within these species, especially noticeable in *C. hominis*, than is seen in industrialized countries (17, 31).

### Genotyping in *Cryptosporidium* Surveillance and Outbreaks

The aim of genotyping in the public health context is to understand transmission and to improve the detection resolution, investigation, and interpretation of waterborne, zoonotic, person-to-person, and foodborne outbreaks. The potential impact lies in:

- Identifying the *Cryptosporidium* species and subtypes that most commonly cause human cryptosporidiosis, and their demographic and temporal-spatial distribution
- Monitoring for the emergence of new species and subtypes in human infection
- Improving detection, investigation, and interpretation of outbreaks
- Increasing the sensitivity of epidemiological investigations to identify links and risk factors, and identify the source of outbreaks and contamination.

In most countries, routine surveillance captures *Cryptosporidium* as an organism, but not species. Where genotyping is used to inform public health, it is mainly in industrialized countries but the framework varies. For example, in England and Wales, clinical diagnostic laboratories have been sending *Cryptosporidium-*positive stools for genotyping for many years, both for molecular surveillance and for outbreak investigations, and most diagnostic stools are genotyped (5, 32). In France, testing for *Cryptosporidium* is not part of routine diagnostic parasitological testing, but a national network of sentinel laboratories was established to test for and genotype new and outbreak cases of cryptosporidiosis (ANOFEL Cryptosporidium National Network, 2010). The Netherlands, Sweden and Scotland also use sentinel laboratories to provide sporadic and outbreak samples for genotyping in reference laboratories (28). In the USA, the Centers for Disease Control and Prevention is developing CryptoNet, a molecular-based surveillance system aimed at the systematic collection and molecular characterization of isolates using 18S rDNA PCR-RFLP and *gp60* sequencing (https://www.cdc.gov/parasites/crypto/cryptonet.html). In Germany, Norway, Spain, Ireland, Northern Ireland, Australia, and New Zealand, *Cryptosporidium* genotyping has been used in epidemiological research projects and/or for supporting outbreak investigations (28, 33, 34), while the focus in Asia, Africa, and South American countries has been on molecular epidemiological research (29, 30, 35).

Molecular surveillance data in the United Kingdom (UK) for example has shown that >95% of cases are caused by *C. hominis* or *C. parvum*. Two seasonal peaks in cases occur, with *C. parvum* consistently causing the majority of cases in spring and *C. hominis* predominating in the autumn peak, with much higher rates of foreign travel also reported during this second period (32, 36–38). A similar temporal pattern has been reported in New Zealand (39), but contrasts with the epidemiology in Ireland, where there is no autumn peak and *C. parvum* predominates all year (33, 40). This is likely due to the highly rural socio-geography of Ireland and the greater potential of zoonotic transmission, a feature also seen in rural regions of Great Britain (36, 38). In the UK, the highest incidence of cryptosporidiosis is in children under 5 years, with a second smaller peak in adults in their 20s and 30s; in England and Wales in the period 2000 to 2003, *C. hominis* predominated in infants and the 30–39 year age group (32), and in children <10 years and adults in the period 2004 to 2006 (37), suggesting transmission between children and caregivers. In Ireland, where *C. parvum* predominates, the adult peak does not appear but this may be a testing bias (33, 40).

Although the sentinel surveillance in France is not wholly representative of the French population due to the structure of the network resulting in the inclusion of a higher proportion of hospitalized cases (70%), particularly over-representing the proportion of HIV-infected patients, certain trends are noticeable (ANOFEL Cryptosporidium National Network, 2010). There appears to only be a late summer/autumn peak each year, but the case numbers per month were too low to determine any species-related seasonality. However, *C. parvum* was more prevalent each year compared to *C. hominis* (54.2 vs. 36.5%) and with the remaining 9.4% representing other species (particularly *C. felis*). The seemingly high number of unusual species were mainly found in the over-represented immunocompromised patients (82.8%), which may explain their higher prevalence than in the UK for example.

In the Netherlands, only an autumn peak in case numbers is present in surveillance data, and the predominant species infecting people does not seem to be stable between years. One study undertaken between 2003 and 2005 reported a higher prevalence of *C. hominis* (70.3%) than *C. parvum* (18.7%), with 9.9% cases having both species, and a single case of *C. felis* (41). The infecting species was significantly associated with patient age, with children (aged 0–9 years) more frequently infected with *C. hominis* and adults (over 25 years old) more frequently with *C. parvum* (41). However, over a 3-year study from April 2013, *C. parvum* was most prevalent in years one and two, but in year three (April 2015 to March 2016) *C. hominis* predominated and cases did not decline toward the winter as they had done in previous years (42). Whether these apparent shifts were a function of fluctuating participation in the sentinel scheme or another reason is not known. In England and Wales apparent shifts have also been seen; from 2000 to 2003 the ratio of *C. parvum*:*C. hominis* nationally was close to 1, but in the period 2004–2006 it was 1:1.5, most noticeable in 2005 when it was 1:2.3 and major *C. hominis* outbreaks may have influenced the distribution (37). The UK and the Netherlands both reported an excess in cases of *C. hominis* with similar epidemiology in the latter part of 2015, and despite *gp60* sequencing identifying subtype IbA10G2 and enhanced surveillance, no explanation was found. This was the second time an international *C. hominis* excess had been reported; in the late summer of 2012 the Netherlands, UK, and Germany reported similarly unexplained increases (43).

In the United States (US) national cryptosporidiosis surveillance through CryptoNet is in its infancy, but there seems to be a high diversity of *Cryptosporidium* species and subtypes causing human cryptosporidiosis compared to other industrialized nations (19). While *C. hominis* and *C. parvum* cause the majority of cases, unusual species such as *C. ubiquitum* and the chipmunk genotype are also seen, particularly in rural areas and may suggest an important role of wildlife in transmission, either directly or through drinking untreated water (19). While general surveillance of *Cryptosporidium* species and genotypes in the US is still fairly new, outbreak surveillance has been carried out for many years through the National Outbreak Reporting System (NORS). Analysis of 444 outbreaks of cryptosporidiosis between 2009 and 2017 demonstrated most were in the autumn and caused mainly by waterborne and person-person transmission (44). Molecular data are available for some of the outbreaks on the NORS website https://wwwn.cdc.gov/norsdashboard/. Genotyping data for 131/178 (74%) outbreaks in the same time period in England and Wales showed 69 were caused by *C. parvum* (which caused all animal and environmental contact and food-borne outbreaks, and a minority of recreational water outbreaks), 60 were caused by *C. hominis* (most of the recreational water and all person-to-person spread outbreaks) and in two outbreaks both species were identified (5). Both *C. parvum* and *C. hominis* caused drinking waterborne outbreaks. *Gp60* sequencing established linkage between cases and suspected sources in nine animal contact, three swimming pool, and one drinking water outbreaks (5). Thus, the public health benefits of identifying infecting species and subtypes lie in the ability to identify and strengthen epidemiologic links between cases, and in indicating possible exposures and sources to inform outbreak management (5). However, the ability to differentiate zoonotic and anthroponotic *C. parvum* routinely in all cases would be useful.

Identification by sequencing has established that unusual species of *Cryptosporidium*, previously considered without zoonotic potential, can infect people. Enhanced surveillance has provided some understanding of the transmission of these infections. In the UK, cases with unusual species often reported zoonotic exposures; contact with unwell pets was a significant association, and in particular, contact with cats was reported by significantly more cases with *C. felis* (45). Genotyping *C. ubiquitum* from patients in the US revealed mainly the rodent-adapted subtype families (XIIb-XIId) in contrast to the UK where infections were mainly the ruminant-adapted XIIa subtype family (19, 46).

The potential for outbreaks is not limited to *C. parvum* and *C. hominis*. In 2007 *Cryptosporidium cuniculus* (previously rabbit genotype) was first identified in a patient during routine molecular surveillance in the UK (47). The following year an investigation into a drinking water quality incident in England established that oocysts detected in treated water were *C. cuniculus*. Soon afterwards, primary and secondary *C. cuniculus* cases appeared in the supplied local population, with the same *gp60* subtype, VaA18 (48). Importantly, matching the *Cryptosporidium* isolated from the drinking water, the remains of a rabbit discovered in a chlorine contact tank, and the case samples provided strong evidence for waterborne transmission. This was the first outbreak reported to have caused cryptosporidiosis where the etiological agent was a species other than *C. parvum* or *C. hominis*, and established *C. cuniculus* as a human pathogen. It re-enforced the importance of protecting water supplies not only from livestock and sewage contamination, but also from wildlife.

Sequencing of the *gp60* gene has identified changes in the circulation of predominant subtypes, and the emergence of virulent subtypes. *C. hominis* IbA10G2 continues to predominate in northern Europe, but in the US in 2007, 40 of 57 sporadic cases from four states were a rare subtype, IaA28R4, with IbA10G2 accounting for just eight cases (49). Since 2013, IaA28R4 has been displaced by IfA12G1R5 as the predominant *C. hominis* genotype in the US associated with both sporadic and outbreak cases (19). In Africa and Asia there is greater variation in *C. hominis* subtypes. For example, in Bangladesh where *C. hominis* is the most common species (>95% of cases) and the seasonality demonstrates a summer peak corresponding to the monsoon, *gp60* analysis revealed 13 different subtypes over a 2 year period (31). Some, for example IaA18R3 and IbA9G3 were present year on year, but other subtypes predominated in some years and disappeared in subsequent years (e.g., IdA15G1 was very common in 2015, but not in 2016 when IaA19R3 and IeA11G3T3 were dominant), indicating a dynamic and frequent transmission (31).

In Europe there is more variation among *C. parvum* than *C. hominis*, although IIaA15G2R1 and IIaA17G1R1 are often (but not always) the most common (5, 19, 50). Genotyping has increased our capacity to detect, investigate and interpret outbreaks. For example, in 2012, *C. parvum* IIaA15G2R1 was used as part of the case definition in an analytical study to investigate a large outbreak (>300 cases) across England and Scotland. A statistically significant association was identified with consumption of pre-cut, bagged mixed salad leaves from a specific national retailer (51). Also in 2012, an outbreak in schoolchildren was associated with a visit to a holiday farm in Norway (52). Genotyping of isolates from cases and potential animal sources on the farm revealed the same rare subtype of *C. parvum*, IIaA19G1R1, in the cases, lambs and goat kids (52). The same holiday farm was also involved in a previous outbreak in 2009 and the same subtype was identified retrospectively, suggesting that in the absence of newly introduced subtypes, existing subtypes can be stable and circulate on the farms for many years (52).

Although *gp60* sequencing has played an important role in refining epidemiological investigations, it is somewhat surprising that there is no standardized multilocus genotyping scheme for *Cryptosporidium* surveillance and outbreaks. Additionally, the lack of suitable markers has hampered our understanding of the main transmission pathway (zoonotic or anthroponotic) of *Cryptosporidium* species and subtypes. As discussed in this paper, genomics has an important role to play in the identification of new markers and the development of a MLG scheme, and the aspiration is that application would eventually become nationally systematic.

### Multilocus Genotyping

Currently multilocus genotyping (MLG) is mainly applied to study the population structure of *Cryptosporidium* spp. with few reports describing its utility in surveillance or outbreaks. One example is an investigation into a Swedish swimming pool outbreak in 2002, where multilocus genotyping revealed two concurrent *C. parvum* outbreaks, with different subtypes linked to the use of either the indoor or outdoor pool, indicating multiple contamination events (53). In England, the epidemiological association of *C. parvum* cases with a drinking water supply was strengthened by MLG (54). However, more often investigations have explored the population structure and biology of *Cryptosporidium*.

In 2015, Widmer and Caccio investigated the relationship between sequence and length polymorphism within a set of biomarkers in the *Cryptosporidium* genome. They compared genetic distances of sequence and length polymorphism, finding that there was a weak correlation between the two distance measures. Their results also indicated that the resolution of *Cryptosporidium* population structure was dependent on the genotyping method used (55). Differences in varying extents of host-associated (56, 57) and geographical segregation (24, 58–60), and the extent of panmixia vs. clonality, depending on the population studied (21), have been reported. For example, in Spain, *C. parvum* in cattle herds was reported to show a panmitic population structure contrasting with sheep where *C. parvum* populations appeared more clonal (19, 61, 62). This may have been a function of the predominance of *C. parvum gp60* subtype family (IId) in sheep in the study region of Northeastern Spain (63) as IId has been reported to be clonal in other regions/countries (64).

Pamixia in *Cryptosporidium* spp. may reflect the increased potential for genetic recombination between more diverse isolates than is available in these supposed clonal populations of parasites. The presence of mixed populations with multiple alleles is the driving force of increased variation and the emergence of new subtypes due to recombination events (65–67). In some studies, for example in Scotland *C. hominis* populations have shown clonality (58), but in a cohort of children in Peru, genetic recombination was detected in some *C. hominis* IbA10G2 samples using MLST of 32 polymorphic loci, despite the overall clonality of the *C. hominis* population (65).

However, with the vast majority of *C. hominis* isolates in many areas, including northern Europe and Australia, demonstrating the dominant IbA10G2 (21) the potential for recombination with other more diverse subtypes may be reduced through lack of exposure in those regions. In contrast, the wide variety of different *C. parvum* subtypes usually present in local geographic areas make mixed populations more likely. This has been suggested in a study of the global population structures of both *Cryptosporidium* species, where samples from Uganda showed similar panmitic population structures, contrasting with *C. hominis* samples from the United Kingdom and *C. parvum* from New Zealand which showed much more clonal population structures (68). The authors suggest that both *C. parvum* and *C. hominis* population structures appear to be shaped by local or host-related factors rather than being species-specific (68). This was borne out by a study in Sweden that applied a nine-locus SNP-based method to differentiate *C. hominis* IbA10G2 and grouped 44 isolates, from 12 countries (including 7 non-European), into 10 MLSTs with known epidemiologically-linked samples clustering together; geographical clustering was not obvious, however the numbers of isolates from each country were small (69). In the USA, the emergence and spread of *C. hominis* IaA28R4 was investigated by sequencing eight loci (67). Of 95 *C. hominis* samples (62 IaA28R4 samples) from four states, the sequence diversity identified two clear sub-populations separated geographically between Ohio and three southwestern states, and suggested that the Ohio subpopulation was a descendant of the subpopulation in the southwestern states. Furthermore, genetic recombination was seen to occur in IaA28R4 isolates and was likely an important factor in its emergence (67), a finding supported by a comparative study of the genome along with the previously dominant IbA10G2 subtype (70).

For disease surveillance and outbreak investigations, there is a need to establish a common multilocus genotyping scheme to track the sources and spread of infection. In a review published in 2012, Robinson and Chalmers reported that different combinations of loci and methods of analysis had been used, with very few groups using comparable loci (27). For public health purposes it is desirable to have consensus to enable cross-boundary comparisons and investigations and track international spread. An initiative funded by EU COST Action FA1408 “A European Network for Foodborne Parasites: Euro-FBP” (http://www.euro-fbp.org) enabled a workshop to be held between 23 scientists and experts in public and animal health from 12 European countries and the USA on *Cryptosporidium* genotyping (71). The participants discussed the need for, and potential directions of, a standardized typing scheme specifically for surveillance and outbreak investigations. There was general agreement that a robust multilocus genotyping scheme should be developed through collaborative laboratory studies, to standardize a method for meaningful interpretation of genotype occurrence and distribution trends, and where possible incorporate into national surveillance programs (71). To achieve this multiple markers spread, sufficiently across the genome, are required. The recent generation of genome data facilitates the identification of markers that show potential to be combined for MLG investigations specifically for surveillance and outbreak investigations (15).

## Whole Genome Sequencing

While we aspire to using WGS routinely in public health investigations of *Cryptosporidium* cases in the way it is applied to some bacterial pathogens (72–74), the reality is that this is still a way off. Direct sequencing would provide timely investigation of public health incidents, but it poses a challenge for this parasite; it is difficult to culture and bioinformatics pipelines have not been validated for public health purposes as *Cryptosporidium* has suffered from lack of prioritization in genomics programs.

The first technical problem is the amount of DNA that is required. Although this varies depending on the technology used, for example, the Nextera XT DNA kits that have been used in several publications require 1 ng of DNA, and as each oocyst contains 40 fg of DNA it means that 2.5 × 10^4^ oocysts are required without losses and in a practical volume (75). To generate sufficient DNA, oocysts may be propagated through animals, but *Cryptosporidium* populations have been shown to change through natural host-based preferential selection of individual subtypes or further recombination into new subtypes. For example, the “isolate” that provided the first reference *C. hominis* genome in 2004 (TU502) was subsequently serially propagated in gnotobiotic pigs over many years resulting in a different subtype in 2012, which was likely due to the original population being overgrown by another contaminating isolate (76). Additionally, the availability of host animals appropriate to the *Cryptosporidium* species in question (Table 1), and the ethics, time and cost resources that are associated with propagation are prohibitive. As propagating oocysts is not a practical solution, obtaining enough clinical sample is the next hurdle, as the volume of stools often submitted is very small. Purity is also a challenge because feces is the starting point, so *Cryptosporidium* DNA is overshadowed by non-target DNA from the biome and host. Lack of purity has been overcome by the combination of several techniques including harvesting by flotation, further purifying by immunomagnetic separation and using the natural chlorine resistance of *Cryptosporidium* oocysts to surface-sterilize them with bleach (75, 77).

The sufficiency of available *Cryptosporidium* DNA has also been addressed through the use of whole genome amplification (WGA) techniques, which now mean that very small amounts of DNA, even from single oocysts, can be used for genome sequencing (77, 78). Guo et al. used WGA to enrich *Cryptosporidium* DNA from six discrete species/genotypes extracted from 24 human and animal fecal samples (77). The results were encouraging, showing that *Cryptosporidium* DNA was significantly enriched, allowing for coverage of > 94% of the genome (77). This ability to whole genome sequence from very low numbers of oocysts is a development that may help when investigating environmental samples and other transmission pathways. Additionally, it may also alleviate problems encountered when whole genome sequencing a mixed population of oocysts. The concern that WGA could result in higher numbers of errors introduced into the genome sequence due to the fidelity of the enzymes used is also unfounded. The presence of four sporozoite genomes in a single oocyst helps, as any errors introduced in the first cycle are unlikely to occur at exactly the same place in more than one genome, so subsequent copies from the other genomes (containing the correct sequence) should overshadow any errors. Although WGS technology has developed and some of the technical hurdles have been overcome to enable direct sequencing (75, 77, 78), we are still not at a point where it can be used to inform in real-time for meaningful surveillance or during outbreak investigations. Aside from technical and resource issues, the lack of user-friendly, validated pipelines specifically designed to generate data in a form that is useful to public health practitioners during the management of incidents, make direct whole genome sequencing currently impractical. Nevertheless, genomic data are being used for biomarker discovery and to understand genetic diversity in parasite populations in different settings. These developments are described below, and arise from the progression of *Cryptosporidium* whole genome sequencing and assembly over the last two decades.

### Progression of Whole Genome Sequencing and Assembly

Attempts to sequence the genome of *Cryptosporidium* began in the early 2000s. Initial attempts involved cloning sheared fragments into plasmid vectors and Sanger sequencing. This approach resulted in > 9x coverage of the genome and yielded a fragmented assembly of 221 contigs of length > 5 kbp (79). A more advanced sequencing project was undertaken to resolve gaps, using large *C. parvum* fragments contained within lambda DASH II libraries, and sequence missing DNA using a primer walk strategy (79). The completed genome of *C. parvum* (Iowa II) along with a preliminary annotation was first published in 2004 by Abrahamsen et al. (80) who passaged oocysts through an animal donor to produce enough parasitic material for the extraction and purification of sufficient amounts of DNA. A random shotgun sequencing approach was used, which yielded a complete genome with coverage of 13x over 18 large contigs (80) and was shortly followed by the publication of the first draft genome of *C. hominis* (TU502) in late 2004. However, this *C. hominis* genome proved to be much more fragmented than that of *C. parvum*, resulting in a sequence consisting of 1,422 contigs (81).

In 2015, the *C. parvum* (Iowa II) reference genome was reassembled and reannotated, and a new *C. hominis* reference genome (UdeA01) published (82). The updated assembly resolved all eight chromosomes from the 18 scaffolds in the previous genome, representing the first chromosome level assembly of *C. parvum*. The reannotation effort increased the number of putative genes from 3807 to 3865 for *C. parvum* Iowa II, and predicted the presence of 3819 genes in *C. hominis* UdeA01 (82). In 2016, Ifeonu et al. reassembled and reannotated the *C. hominis* TU502 genome, along with producing new draft genomes of human isolated *C. hominis* (UKH1) and *C. meleagridis* (UKMEL1) along with the avian species *Cryptosporidium baileyi* (TAMU-09Q1) (83). The *C. hominis* TU502 genome proved to be a considerable improvement on the previous 2004 version, being much more complete, and reducing the number of contigs down to 119. Annotation was facilitated by the RNAseq data generated from the oocyst stage of both *C. hominis* and *C. baileyi*, predicting the presence of 3745 protein coding genes in *C. hominis* TU502 and 3765 in *C. hominis* UKH1 (83).

As can be seen in Table 2, there is little difference between the genomes of *C. parvum* and *C. hominis*. They exhibit 95–97% DNA sequence identity; with 11 protein-coding sequences identified only in *C. hominis* and 5 in *C. parvum*, and no large indels or rearrangements apparent (84). The high conservation in the *C. hominis* genomes generated from European samples compared to the much more polymorphic *C. parvum* does not appear to be expressed in general observations on structure and base representation as illustrated in Table 2, suggesting that phenotypic differences are potentially due to more subtle sequence divergence (SNPs and Indels) and gene expression. This further illustrates the importance of large-scale sequence comparison of *Cryptosporidium* species to elucidate potentially exploitable variation. Widmer et al. identified a number of highly divergent genes by comparison of the genomes of *C. parvum gp60* subtype IIc and the Iowa II reference (85). Further investigation reveals that genomic evolution was largely reductive, resulting in *Cryptosporidium* depending mainly on host cells for basic nutrients (86).

**Table 2:** The progression of *C. hominis* and *C. parvum* whole genome assembly from initial attempts in 2004 to the completed genomes in 2015 and 2016 (80–83).

**Feature**	***C. parvum* Iowa II (2004)**	***C. hominis* TU502 (2004)**	***C. hominis* UdeA01 (2015)**	***C. parvum* Iowa II (2015)**	***C. hominis* TU502 (2016)**
Genome length	9.10 Mbp	9.16 Mbp	9.05 Mbp	9.10 Mbp	9.10 Mbp
Coding genes (% genome)	3807 (75.3%)	3994 (69%)	3819 (75.4%)	3865 (75.7%)	3745 (77.8%)
GC content	0.3	0.32	0.32	0.32	0.3
Introns	0.05	0.05-0.20	0.109	0.108	not reported
Fragments	18	1422	8	8	119

As more genomes are becoming available at an ever-increasing rate, researchers are able to explore further the biology and evolution of *Cryptosporidium*. Recently, Nader et al. (87) used 21 whole genome sequences to show the existence of two subspecies lineages of *C. parvum* (*C. parvum parvum* and *C. parvum anthroponosum*) with different host-adapted infectivity. Additionally, they identified some of the historic genetic exchanges that have occurred between these lineages and *C. hominis* during the evolution of these different species and subspecies, even suggesting rough time-lines for when these events occurred (87, 88).

In an important epidemiological development, Gilchrist et al. (31) used the methods described by Hadfield et al. (75), to study the genetic diversity of *C. hominis* in slum dwelling infants in Dhaka, Bangladesh, over a 2-year period. As mentioned above, they found that *C. hominis* was more abundant during the monsoon periods and showed high levels of diversity at *gp60* locus. Furthermore, WGS revealed extensive SNP diversity, and very high levels of variation at seven distinct loci. They also detected high levels of recombination within the *C. hominis* populations, evidenced by linkage disequilibrium decay. The genetic diversity of *C. hominis* encountered in the Bangladesh study was found to be far greater than that seen in northern Europe, where the predominant *C. hominis* IbA10G2 subtype is highly conserved at the genome level (50, 71). This study reveals the importance of high-throughput, wide scale genomic sequencing and analysis in elucidating the complex population structure of the parasite worldwide (31).

In another study, WGS was also used for a comparative genomic analysis between two subtypes of *C. hominis* that have been dominant in the US at various times, IbA10G2 and IaA28R4, and *C. parvum* (70). Their genome comparison revealed evidence of genetic recombination in the two *C. hominis* subtypes, and also some unique genetic differences between *C. hominis* and *C. parvum*, and multigene families that may contribute to the host variation between these two species (70).

### Genome Availability

The advent of the new techniques to facilitate the DNA extraction, enrichment, sequencing, and assembly of high quality *Cryptosporidium* genomes from clinical samples, provides an opportunity to greatly expand the number of genomes available. An EU funded collaboration (Aquavalens project, www.aquavalens.org) between several institutions generated 27 assemblies of *C. parvum, C. hominis, Cryptosporidium viatorum, C. ubiquitum, C. cuniculus*, and *C. meleagridis* directly from clinical isolates using the DNA extraction and purification protocol described by Hadfield et al. (75) and Nader et al. (87). Under another EU funded project, COMPARE (https://www.compare-europe.eu/), 31 new *C. parvum* and 19 new *C. hominis* genome assemblies were generated from clinical isolates, using the DNA extraction and purification protocol described by Hadfield et al. (75), and the DNA enrichment protocol described by Guo et al. (77). A further 14 *C. hominis* genomes, representing 9 different *gp60* subtypes, have also been published (89) and are available as a Bioproject (PRJNA307563) on the National Center for Biotechnology Information (NCBI) online databases. Currently, whole genome assemblies of isolates from human and animal derived *Cryptosporidium* spanning 9 species, are available as Bioprojects on NCBI databases (see Table 1), but this number is rapidly increasing as methods and technology become more available. The *Cryptosporidium* genomics resource CryptoDB (http://cryptodb.org/), provides access to species including *C. hominis, C. parvum*, other zoonotic species including *C. meleagridis*, and host-adapted species rarely found in humans (*Cryptosporidium muris, Cryptosporidium andersoni, C. baileyi*, and *Cryptosporidium tyzzeri*) and provides analytical tools to mine and compare the genomes sequences and their functionality (90, 91). A number of unassembled, unprocessed raw read sequences are also publically available via online repositories such as GenBank and the Welcome Trust Sanger Institute FTP servers.

### Sequencing Using Long-Read Technology

Recently, there have been attempts to generate *Cryptosporidium* sequences using long-read technology, such as MinION by Oxford Nanopore, and Pacific Biosciences. There exist a few draft genomes from long reads generated by PacBio, but most are yet unpublished. However, a *C. parvum* PacBio sequence is available on the Welcome Trust Sanger Institute FTP servers (ftp://ftp.sanger.ac.uk/project/pathogens/Cryptosporidium) that was generated to map shorter Illumina reads to during the study in Dhaka that explored the genetic diversity of *C. hominis* (31). Currently, there have been no successful attempts at sequencing the genome using the MinION platform published. This is likely due to the large amount of DNA required to generate such reads using this particular technology, which is a known difficulty associated with *Cryptosporidium* genomic sequencing.

### Pitfalls in Genome Assembly

**Figure 3:**
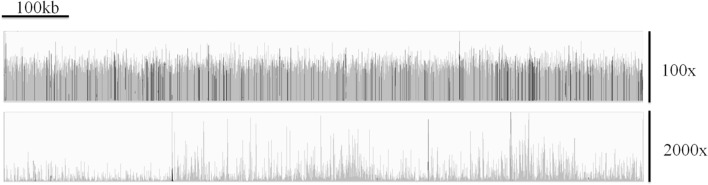
A comparison of the coverage over chromosome 1 *of C. parvum* Iowa II (top track) and the clinical isolate UKP3 (bottom track), showing the highly uneven coverage typically exhibited from many clinical isolates. Reads were mapped using Bowtie v2.3.3.1 (93) and visualized using Integrative Genomics Viewer v2.4.16 (94).

Morris et al. have outlined difficulties associated with generating reliable and accurate genome assemblies from clinical isolates of *Cryptosporidium* (92). They demonstrated that the issues surrounding extracting sufficient DNA from clinical isolates resulted in highly uneven depth of coverage across the genome (for an example, see Figure 3) which can be seen in sequences generated from clinical isolates by a number of research teams. This, in tandem with the large number of low complexity regions within the *Cryptosporidium* genome, results in widespread genome misassembly when using the Spades assembler (95). Peng et al. further proposed an approach to generating reliable draft assemblies from clinical samples, and demonstrated how accurate resolution of low complexity regions are essential for biomarker discovery using the Iterative De-Bruijn Assembler (IDBA) (96).

Assembly of *C. parvum* and *C. hominis* is facilitated by high quality reference sequences (*C. parvum* IowaII and *C. hominis* UdeA01) which allow for reference-guided assembly. This, however, is not the case for other species of *Cryptosporidium*. It is therefore important to consider whether a reference guided assembly should be attempted, and what reference genome to use. The application of an inappropriate reference sequence may result genome assembly errors.

## Applications, Future Issues, and Research Directions

With the recent expansion in the number of available raw read archives and genome assemblies generated from clinical samples, further *in silico* investigation can be carried out in an attempt to resolve a number of biological questions, such as:

- Can biomarkers differentiate genetic lineages of *Cryptosporidium* spp. virulence or pathogenicity, and therefore act as targets for diagnostic interrogation or novel therapeutics?
- How much variability exists within intergenic regions in species of *Cryptosporidium*?
- To what extent do multiple sub-populations of *Cryptosporidium* spp. exist within an infected host and in single clinical samples and impact of these during onward transmission and even the evolution of the parasite?

### Biomarker Discovery and Analysis

The state of *Cryptosporidium* genotyping is far from resolved, and there is still a large amount of work to be done regarding the discovery, assessment, and selection of suitable biomarkers and genotyping conventions. Subsequent to the increasing availability of genomes is a bottle-neck in the analysis of these data, and there is a need to develop time-efficient, computationally inexpensive and high-throughput (automated) methods of genome analysis. “In house” pipelines have been used for biomarker detection and analysis. A typical example was reported by Perez-Cordon et al. (15), who used Tandem Repeats Finder (TRF) (97) to detect Variable Number Tandem Repeat (VNTR) regions within the genome of *Cryptosporidium parvum* Iowa II isolate and aligned them to homologues within a dataset of genomes generated by Hadfield et al. (75). This pipeline consisted of three primary steps:

1. Tandem Repeat (TR) identification in a reference genome.
2. Discovery of the TR's around the genome of a dataset of assembled genomes.
3. Assessment of these TR's for variation and subsequent viability as Biomarkers.

Using this pipeline, bioinformatic analysis of the Hadfield dataset alone has yielded a large number of novel VNTR regions (15), some of which compare favorably to the commonly used *gp60* marker in their ability to resolve discrete subtypes of *C. parvum*. Automating pipelines, can utilize the increasing amounts of whole genome sequence data available for *Cryptosporidium* allowing for the discovery of novel VNTRs in a high-throughput manner.

In addition to novel VNTR markers, genome analysis of other *Cryptosporidium* species and genotypes can allow for the redescription of known markers in these for the development of new subtyping tools. One example, is with the zoonotic species *Cryptosporidium ubiquitum*, where the homolog of *gp60* was diverse from those of *C. hominis* and *C. parvum* so could not be used to differentiate isolates (46). Li et al. used whole genome sequence data to identify and develop a *gp60* subtyping tool that allowed the differentiation and showed apparent host-adaptation (46). Another example, described the development from whole genome sequencing data of a two marker subtyping tool (*gp60* and a mucin protein gene) for the zoonotic chipmunk genotype I (98).

When developing genotyping assays, it is important that biomarkers are selected so as not to influence the outcome of the analysis. For example, markers must be distant enough from each other on the same chromosome or spread over the eight chromosomes to ensure genetic linkage does not occur, and markers must give high enough discrimination when combined to be appropriate for the application in question, such as demonstrating epidemiological relationships (27, 84).

### Multiplicity of Infection in *Cryptosporidium*

It is both biologically plausible (due to unrestricted sexual recombination between sub-populations), and there is strong evidence (described below) that infections can arise from, and give rise to, multiple sub-populations of *Cryptosporidium* spp. which will be present in individual hosts (termed here multiplicity of infection—MOI) and thus clinical samples. This is driven by meiotic division in the zygote resulting in potential re-assortment of chromosomes (Figure 2b). As a result, the genomes of the haploid sporozoites within an oocyst may differ from each other and the parent sporozoites. Grinberg and Widmer demonstrated the common occurrence of MOI and provided evidence that the degree of MOI may depend on prevailing transmission patterns within geographical regions (25). The current approaches of Sanger sequencing results in the resolution of a single allele at each locus for the population, which, if MOI is present, would in effect simply represent the most populous sequence variant at each locus within the assembly. Grinberg and Widmer illustrated this from three hypothetical infections (25), but the potential extent for MOI is theoretically even greater (Figure 2b). This may confound epidemiological analysis, which generally relies on the assumption that large-scale genetic recombination does not occur within a host, and that a single host exhibits a single, clonal population. Furthermore, it has been suggested that MOI is a driving force behind the evolution of virulence, and has a complex relationship with both the virulence experienced by the host, and transmission (99, 100). It is therefore essential that MOI is well-understood and accounted for in order to develop novel prevention strategies in the fight against cryptosporidiosis and other parasitic diseases. The investigation into the impact of MOI relies on the accurate and reliable detection and discrimination of discrete populations of parasites, not readily achieved by current genotyping approaches. There are a few major alternatives to achieve this:

- Cloning and sequencing key loci to detect variation.
- Isolating and sequencing single oocysts from clinical samples.
- Comparing length polymorphism at multiple loci.
- Investigating sequence variation among reads within short read archives generated by Next Generation Sequencing (NGS).

These approaches investigate MOI from very different angles: variable locus cloning and single cell sequences from an experimental angle, and length polymorphism and sequence variation within reads from an *in silico* angle. This lends them unique challenges to overcome. By cloning PCR amplicons of selected loci (*gp60* and hsp70) and utilizing Next Generation Sequencing (NGS), Grinberg et al. reported the presence of numerous sub-populations within single isolates of *C. parvum*. They demonstrated the presence of two *hsp70* and 10 *gp60* alleles within their two isolate dataset. Furthermore, they reported that in both isolates there was a dominant allele, which represented the majority of the amplicons sequenced (101). Single oocysts were isolated and sequenced by Troell et al. (78) with a view to elucidate these putative intra-isolate sub-populations. Sequencing 10 oocysts individually resulted in assemblies of 49.4–91.8% of the size of the *C. parvum* Iowa II reference genome. By pooling the reads from all 10 oocysts, they generated a 94.4% complete genome. Variation at multiple loci was detected between the assembled genomes, verifying the presence of discrete populations within the “isolate” (78). Analysis of fragment length polymorphism can highlight MOI, however, due to PCR-based amplification of the fragments, minority variants are largely undetectable (25). To compare the results obtained from Sanger sequencing and NGS, Zahedi et al. investigated *gp60* amplicons from 11 *C. hominis*, 22 *C. parvum*, and 8 *C. cuniculus* animal samples from Australia and China (102). They demonstrated that NGS is more effective at resolving the presence of multiple populations of *Cryptosporidium* within a sample, and the extent of MOI. There was concordance between the subtypes identified by both platforms, but additional subtypes were identified using NGS on *C. parvum* and *C. cuniculus gp60* amplicons, but not *C. hominis*.

The major issue with the experimental approaches detailed above is that they are expensive, extremely labor intensive and time consuming, leading to poor scalability. This leads to a major problem in generating sufficient data with which to begin to unravel the role of these parasite sub-populations, and to understand their overall impact on global public health. It is expected that they will have roles in affecting transmission by reducing host-fitness (virulence), and in generating novel subtypes via sexual recombination. There is therefore a great need to develop strategies which allow us to carry out investigations in a high-throughput manner, utilizing the wealth of raw genomic data is available for *Cryptosporidium* and other related parasites. Using biomarkers discovered from the analysis of the increasing number of high quality genomes, the opportunity arises to start to investigate MOI using *in silico* techniques, by mining raw read sets sequenced from clinical samples for information, which may have been previously unattainable. This approach involves three stages:

1. Identification of target regions for read interrogation. It is essential to select target regions, which are likely to show variation in-host, and it is therefore wise to select loci which show large amounts of variation between hosts.
2. Identification of reads within a single-host read set which have captured the target region.
3. Assessment of variation of the target sequence amongst reads which were identified in step 2.

**Figure 4:**
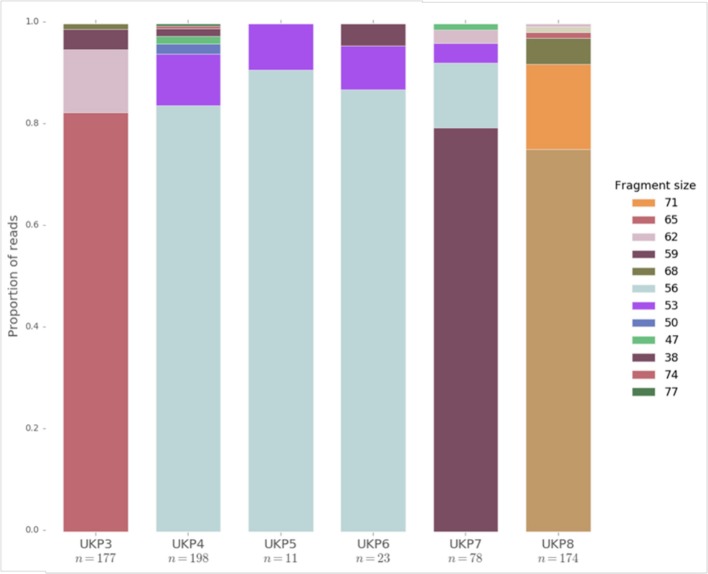
The distribution of fragment lengths at the *gp60* locus mined from raw read sets generated from human clinical samples of UK isolated *C. parvum* by Hadfield et al. (75). Fragment lengths are given in the legend. *n* refers to the number of reads which fully captured the *gp60* region, and are therefore presented in the data.

A high level of variation within a single-host read set indicates the presence of multiple populations. Preliminary analysis of the Hadfield et al. dataset (75) indicated extensive variation at multiple tandem repeat loci around the *Cryptosporidium* genome, indicating highly complex in-host population structure. Results for variance mining at the *gp60* locus can be seen in Figure 4, which shows high levels of fragment length variation. However, there is invariably a single allele which appears to be most frequently exhibited within reads, and therefore considered dominant. This is in agreement with the findings reported by others, which show similar population structure (78, 101). There is, however, a disparity in the extent of MOI in *Cryptosporidium* between laboratory evidence by fragment sizing of key loci, and by mining NGS data. This is potentially due to the limited sensitivity of such approaches to identify multiple alleles of similar fragment size. Furthermore, PCR may preferentially amplify more abundant alleles, resulting in the less abundant alleles being obscured, as shown by Grinberg et al. who initially only identified the predominant alleles in their samples by PCR and Sanger sequencing (101). It may also be the case that such studies were not designed to detect multiple alleles within a single sample, and therefore underestimate the incidence of MOI. Consequently, care should be taken when interpreting entirely *in silico* results in the absence of experimental data. Due to MOI being a new area of investigation in *Cryptosporidium* research, the reliability of *in silico* approaches to elucidate in-host population diversity is still unclear, particularly in the light of studies indicating extensive contamination of samples (77). Preliminary results, however, appear to make predictions which are in accordance with experimental and epidemiological evidence, giving confidence in such data.

Natural transmission studies from analyzing secondary infections and those in farm settings has shown that dominant subtypes can be stable for many years or they can vary from year to year. For example, the outbreaks among visiting children on a holiday farm in Norway showed the same *gp60* subtype, IIaA19G1R1, was still circulating over several years and an investigation into secondary transmission within households after the children returned home also found the same subtype (103). While there was no evidence at the *gp60* gene of mixed populations in this example, in farm settings it is common for multiple subtypes to be present (104, 105). During a study of household transmission in a rural and urban setting in Bangladesh, a wide variety of *gp60* subtypes were found, particularly in the urban setting, but often there were concurrent infections with the same subtype within households and therefore it was mostly impossible to know the directionality of transmission (106). Where there were different subtypes within households it is unclear whether these stemmed from external sources rather than secondary transmission within the household (106). However, despite these studies there is a lack of data from mixed natural infections and the changes or dominance of subtypes that may occur during onward transmission, something that warrants further investigation using multilocus tools or whole genome data. Cama et al. used MLST to characterize differences in Iowa reference *C. parvum* isolates that had been maintained in different laboratories and described differences that were likely the results of passages through calves infected with exogenous *C. parvum* (107). This genetic drift in reference isolates was also seen with the TU502 reference *C. hominis* isolate between 2005 and 2012 following multiple animal passages (76). Therefore, the implications of MOI for surveillance and outbreak investigations are uncertain. As drift may happen in the longer term but not necessarily in the short term, detecting an outbreak “type” is reasonable, but equally it could be that two cases with apparently different subtypes are still actually linked if there is bias in the detection of dominant alleles.

## Conclusions

WGS holds tantalizing promise for better understanding the transmission of cryptosporidosis, but there are still good reasons as to why it is not used routinely for diagnostics in a clinical setting. These include issues with extracting high quality pure DNA from clinical samples and issues with uneven depth of read coverage that leads to gaps in the assembled genome sequence. This later issue has important implications for cost: reducing costs by sequencing at a low depth of coverage is problematic, because it will increase the size and frequency of gaps in the assembled genome sequences. Nonetheless, while WGS is not yet on the horizon as method for routine clinical genotyping, it is indirectly having an important influence on clinical diagnostics. For instance, WGS is being used to guide and inform the development of MLST schemes, such as those based on VNTRs and fragment sizing. It is providing key insights into the evolutionary development of *Cryptosporidium*, including the discovery of new subspecies. Perhaps most important in terms of understanding the transmission of the disease, WGS is providing key insights into MOI. While evidence for MOI is occasionally found using fragment sizing, preliminary WGS analysis shows that is it is much more prevalent than the evidence from fragment sizing might suggest. WGS shows that although clinical samples do indeed contain multiple alleles, a single highly abundant allele usually dominates the data sets. It is highly likely that only the dominant allele that is detected via fragment sizing, with the other alleles remaining undetected. Resolution of these multiple populations is a stepping-stone to understanding the driving factors behind the evolution of virulence, and how new subtypes and genotypes arise in *Cryptosporidium*.

## Author Contributions

RC devised and revised the manuscript. GR, AM, and MS drafted the manuscript. All authors approved the final manuscript.

### Conflict of Interest

The authors declare that the research was conducted in the absence of any commercial or financial relationships that could be construed as a potential conflict of interest.
